# Exploring Intensive Care Nurses' Experiences and Perceptions of Patients With a Co‐Morbid Mental Health Disorder

**DOI:** 10.1111/nicc.70437

**Published:** 2026-03-09

**Authors:** Angela Teece, John Baker

**Affiliations:** ^1^ School of Healthcare University of Leeds Leeds UK

**Keywords:** critical care, mental health, nursing, self‐harm

## Abstract

**Background:**

Patients with a co‐morbid MH disorder are roughly twice as prevalent in intensive care units (ICU) than in other general secondary care areas. Such patients may be subject to social stigma and diagnostic overshadowing while being nursed in general healthcare settings. Studies in non‐ICU areas suggest that staff lack knowledge and work in a non‐collaborative manner, impacting negatively on the delivery of holistic humanised care. There is limited evidence on this topic from the perspective of ICU nurses.

**Aim:**

This study aimed to add to the small body of evidence exploring how ICU nurses experience caring for patients with an MH disorder.

**Study Design:**

A pragmatic approach was taken. Twelve members of registered ICU nursing and advanced practice staff were recruited via social media and professional networks. Participants engaged in online individual semi‐structured interviews which were audio‐recorded. The transcripts were checked and then analysed using a six‐stage reflexive thematic analysis process.

**Findings:**

Five themes were identified: (1) frustration and futility, (2) the impact of a lack of knowledge, (3) perceptions, (4) unpredictable and violent and (5) the ICU environment is unsuitable.

**Conclusions:**

The findings of this study indicate that ICU nurses require additional support, education and clinical leadership to manage patients with an MH disorder. Participants expressed feelings of frustration regarding patients' limited access to on‐going MH support. Participants appeared especially uncomfortable when caring for patients who had self‐harmed or attempted suicide. A need for further research into the experience of MH patients in ICU is indicated.

**Relevance to Clinical Practice:**

Due to the high prevalence of patients with an MH disorder in ICU, it is important that ICU nurses understand their specific needs and how socially held stigmatised views can impact on care. Stigma and previous traumatic events can lead to staff avoiding engagement with MH patients. Strong clinical leadership, collaborative working and continued education are necessary to support both staff and patients.

## Impact Statements

**What is known about this topic?:**

- The prevalence of mental health disorders in ICU patients is twice that of patients in other areas of secondary care.
- There is a paucity of high‐quality research in this area of practice. Previous reviews suggested that nurses may deliver care influenced by socially generated bias and stigma.
- Nurses working in ICU appear to lack confidence in their skills when managing patients with mental health disorders. A need for greater support and education was identified.

**What this paper adds:**

- ICU nurses feel unsupported by MH services and experience frustration at the assumption that ICU is a ‘safe place’ due to its capacity for 1:1 nursing. They feel the area is unsafe for this patient group, and there were concerns that they might harm other patients.
- ICU nurses are keen to deliver high‐quality patient care, but feel concerned about their ever‐widening nursing role and the ability to maintain skills.
- This is an important topic and participants were keen to share their experiences. More support for clinical staff is indicated.
- This study is part of a series of work undertaken to explore the experiences of ICU nursing staff when caring for a patient with a co‐morbid MH disorder. The study has demonstrated a need for further support and education and a lack of integration between mental and physical health care.

## 1 Introduction

This study aimed to explore how UK‐based registered nurses and Advanced Critical Care Practitioners perceive and experience patients with a co‐morbid mental health (MH) disorder who are admitted to a non‐psychiatric intensive care unit (ICU).

Patients with a co‐morbid MH disorder are roughly twice as prevalent in ICUs than general wards in secondary care hospitals [1], but there is a paucity of evidence regarding how staff perceive and manage this patient group. Clinical outcomes for this group have been studied in non‐ICU areas. Patients with a severe mental illness, such as major depression, personality disorder or schizophrenia, experience a significantly higher mortality rate than those without a MH disorder [2, 3]. A German study identified that adults aged over 70 with an MH co‐morbidity experienced higher rates of in‐hospital mortality than those without an MH disorder [4]. Persons with an MH disorder are known to experience stigma and exclusion, which can inhibit their access to health services and challenge their ability to understand and communicate their needs [5, 6, 7, 8]. This is exacerbated by diagnostic overshadowing, where the MH diagnosis overshadows physiological issues and the patient as a person [8]. Post‐operative patients with an MH disorder are more likely to require an ICU admission and have a prolonged hospital stay, both of which incur considerable additional healthcare costs [9].

General hospital staff can exhibit negative attitudes and avoidance, particularly towards people who engage in deliberate self‐harm or attempt suicide. These impact negatively on patient experience and contribute to adverse patient safety events through biased decision‐making [10, 11, 12]. Nurses have also reported feelings of fear and futility when caring for patients with an MH disorder [13] which can lead to burnout [14], alongside concerns that they lack the appropriate skills to care for this cohort [13].

An integrative review [15] found a paucity of evidence regarding how ICU patients with a co‐morbid MH disorder were perceived by nurses. Additionally, the review identified themes which demonstrated the use of stigmatising language to ‘other’ this patient cohort, reducing them to a homogenous mass, alongside assumptions about violence and aggression which led to an increased use of restraint and a feeling that ‘they’ do not belong in ICU. There appeared to be an appetite for increased staff education and support, but participants were unclear about how this should be delivered [15]. A scoping review from the same year [16] found similar themes and identified the need to support ICU nurses in providing holistic and safe care to enhance the well‐being of this patient group.

There is minimal evidence that explores this topic from a UK‐based perspective. Five of the eight studies included in one integrative review originated from Australia [15]. Soreny [17] interviewed four nurses working within a neuroscience ICU who described feeling under‐skilled when managing patients with an MH disorder. Patients with an MH disorder who are nursed in ICU are a vulnerable group who require skilled collaborative care, delivered by nurses who are supported to provide unbiased and humanised care. This study aimed to further develop the existing knowledge on how ICU nurses experience the care of a patient with a co‐morbid MH disorder.

## 2 Design and Methods

This study aimed to add to the small body of evidence exploring how ICU nurses experience caring for patients with an MH disorder. To achieve this, Registered Nurses and Advanced Practitioners were recruited to undertake semi‐structured interviews. This study employed a pragmatic approach. Virtual semi‐structured individual interviews were undertaken by Author A (female, ex‐ICU nurse). An interview schedule was developed to help the researcher and participant to maintain focus on the topic. Three written vignettes were prepared to stimulate discussion if required (Table 1). The vignettes were reviewed by an expert through experience group hosted by the authors' institution. This group of service users and relatives had experience of both ICU and MH care. Participants were encouraged to reflect on their own clinical experiences and storytelling was embraced.

**TABLE 1:** Vignettes created for interviews.

Patient admitted to critical care with polypharmacy overdose and multiple previous overdoses. Known to have personality disorder currently intubated and ventilated. Plan to allow to wake and extubate today. Agitated and anxious on previous admissions.
2 Patient admitted with sepsis and community acquired pneumonia. Patient is obese and has bi‐polar disorder. Normally supported by the community MH team. Now weaning via tracheostomy.
3 Male admitted following collapse at home following drug use. Treated for rhabdomyolysis now awake and self‐ventilating. Known anxiety and depression. Has not accessed MH support previously.

### 2.1 Setting and Sample

Twelve members of UK‐based ICU staff were recruited. Participants were eligible for inclusion if they were Registered Nurses or Advanced Practitioners working in adult critical care with experience of caring for patients with a co‐morbid MH disorder. Non‐registered, allied health professionals and medical staff were excluded. Participants included staff nurses (*n* = 4), of whom *n* = 1 was dual registered (MH and adult), sister/charge nurses (*n* = 3), outreach nurse (*n* = 1), matron (*n* = 1), trainee advanced critical care practitioners with a nursing background (*n* = 2) and an educator (*n* = 1). Recruitment used a snowballing approach and was undertaken via social media (Facebook, X, LinkedIn and Bluesky) and professional networks between July and September 2025. Potential participants contacted Author A to express interest and were provided with further details about the study. A remote interview was scheduled with Author A if they decided to join the study. No participants withdrew their data from the study.

### 2.2 Data Collection

On recruitment, participants were provided with an electronic copy of the consent sheet which provided details of confidentiality and data storage. All data would remain confidential unless a risk of harm was disclosed. Verbal consent was taken and recorded prior to the interview beginning. Participants were assigned pseudonyms. The aim of the study was reiterated to participants prior to the interview beginning. The interview schedule was followed which aimed to guide participants in talking about their experience with managing patients with a MH disorder. The prepared vignettes were used if participants were felt to be struggling to identify examples themselves.

Interviews ranged from 30 min to 1 h. All interviews were undertaken via Microsoft Teams and were recorded and transcribed by the application. Malterud, Siersma and Guassora [18] suggest the idea of recruitment until information power is achieved. A small participant group, who hold experiences relevant to the focus of the study, will be more able to achieve information power than a larger, less specific sample. In this study, the participants possessed characteristics highly specific to the study aim. This approach encourages the researcher to reflect on the richness of the data they collect and its appropriateness to the study aim and thus estimate when recruitment is complete [18].

### 2.3 Data Analysis

A six‐stage reflexive thematic analysis was undertaken [19]. Trustworthiness was promoted through following this process and accurately recording decisions made throughout analysis and maintaining a research diary. In reflexive thematic analysis, the authors' experiences and inherent subjectivity are valued and ensured through their reflexive engagement with the data and the analytical process [20]. Author A has a background in adult ICU nursing, and an interest in the management of vulnerable groups in mainstream health care. She had previously undertaken semi‐structured remote interviews with critical care staff on a sensitive topic. Author B has expertise in MH nursing, restrictive practices and undertaking pragmatic research projects. Both authors currently work in an academic environment. The authors communicated regularly whilst data collection and analysis were undertaken.

The transcriptions provided by the Teams application were checked by author A for accuracy. Data familiarisation is the process of iterative reading through which the researcher becomes immersed in the world of the data. Coding was the third step and was undertaken using Nvivo software. Aspects of the transcribed data were identified that could be used to answer the research question [19]. Groups of codes with shared meanings formed candidate themes, which were reviewed through the analysis process by both authors. Five themes were finally identified and agreed upon by both authors. The final part of analysis is the process of writing through which the themes are presented and interpreted in the context of the research question [19]. The themes and codes are shown in Table 2.

**TABLE 2:** Summary of themes and codes.

Theme	Codes	Example excerpt
Frustration and futility	We do not understand each other's roles. Lack of unified services Systemic funding issues Support post‐discharge Poor psychiatric provision Lack of MH support on ICU Frustration Conflict between MH and adult services and staff	*They're not helping with this, that and the other like, what are they even doing here? (P1)* Coded to: We don't understand each other's roles, Lack of MH support on ICU, and Conflict.
The impact of a lack of knowledge	What is the right thing to say? We have transferable skills. We do not get enough MH training. MH is too big a topic area. A challenge to maintain skills Densensitisation and avoidance	*Get them like a rugby ball. Pass it on dead quick you know, I mean to someone else without getting involved in it, because I can't fix this. (P10)* Coded to: Desensitisation and avoidance.
Perceptions of patients with an MH disorder	Unpopular and time‐consuming Stigmatised language MH patients are just as sick as an ICU patient. Just like any other patient Empathy and understanding They do it on purpose. Challenging and complex	*This person is not taking the piss. They are being tortured emotionally, psychologically, circumstantially, and I need you to understand that this is their way of dealing with indescribable pain. (P11)* Coded to: MH patients are just as sick as an ICU patient and empathy and understanding.
Unpredictable and violent	Unpredictable A risk to patient and staff safety Need close supervision Need to plan ahead Managing violence Lack of clinical guidance Absence of 24 h support Can we intervene? Control	*It was scary. The fact that we had security guards, we had nursing staff pinning this patient down. We assaulted this patient, in want of better the word, for his own benefit because he was unsafe and he could have killed himself in that state. (P3)* Coded to: unpredictable, managing violence and control
The ICU environment is unsuitable.	The environment is unsafe. Frightening and traumatic No man's land We like to make people better.	*Watching other people come in extremely unwell, intubated and ventilated, you know, with curtains around, but privy to bleeding, screaming, crash calls at 4:00 AM, and if this person already had really significant distress, kind of essentially, in their experience, holding them captive in hospital in witnessing horrible things (P11)* Coded to: frightening and traumatic

### 2.4 Ethical and Institutional Approvals

This study was given favourable review by the University of Leeds School of Healthcare Research Ethics Committee (id: 2755) in May 2025.

## 3 Findings

Five themes were identified from the analysis of the data: (1) frustration and futility, (2) the impact of a lack of knowledge, (3) perceptions, (4) unpredictable and violent and (5) the ICU environment is unsuitable.

**Theme 1**: Frustration and futility: ‘…and it felt like being a person in a big game that isn't actually helping anyone’.

Participants described a disconnection between themselves and MH nurses, referring to them as *different breed* (P2). There appeared to be an expectation that MH support workers allocated to observe patients would assist with care:

They're not helping with this, that and the other like, what are they even doing here? (P1).

This caused resentment because ICU nurses were expected to manage mental and physiological health, while MH nurses only worked within their specific sphere:

*…*you'd never expect a mental health nurse to look after an intensive care patient. (P4).

Some MH professionals who visited patients on ICU were viewed as avoiding undertaking mental health assessment through spurious reasons, for example, deeming patients receiving humidified oxygen via a mask as not fit for assessment (P4) or the misplaced belief that all ICU patients were *drugged up* (P6). Assessments were also criticised for being overly brief:

He needed that person to come and speak to him. They turned up hours later, hours later and just quickly said to me how they're doing. And I gave him a bit of an update and then they were walking off (P12).

Participants expressed concern regarding the level of specialised MH support that patients received in ICU and following discharge. They linked this to systemic funding issues. The poor provision of support within the wider hospital also engendered feelings of frustration and futility:

There's a lack of mental health services, the lack of mental health inpatient beds, the lack of crossover. It's so futile that no matter what you say, you're never going to get the result that you want (P4).

Participants described repeatedly chasing services for support and being *fobbed off* (P11). Patients were observed to self‐discharge without assessment and community support, leading to fears that they would be re‐admitted following an escalation of self‐harm or, ultimately, ‘just going to be found dead’ (P1).

**Theme 2**: The impact of a lack of knowledge: ‘we're just not equipped’.

Participants felt that they lacked theoretical knowledge and practical skills, such as physical restraint training. There was a tendency to ‘step back and get somebody that understands this’ (P7), rather than to attempt to engage with MH patients, whose care one participant described as *a minefield* (P7). ICU nurses felt that they did not know the ‘right thing to say’ to patients admitted following deliberate self‐harm or attempted suicide.

Bad luck, you know, just say bad luck. Oh, yeah, you survived. And they didn't want to survive. So what do you say then? You know, hard luck. You didn't do it this time. (P10).

One participant described a patient in *anguish* (P11) because their suicide attempt had been interrupted. Such situations were described as *emotionally draining* (P1) and *torturous* (P4) over the course of a long shift. The intensity had a profound impact on nurses and, sometimes, led to desensitisation and avoidance:

Get them like a rugby ball. Pass it on dead quick you know, I mean to someone else without getting involved in it, because I can't fix this. (P10).

Participants also described the difficulty in maintaining continuity of care and building relationships while safeguarding themselves from burnout. However, some participants noted that ICU nurses have transferable skills which can be used effectively with this patient cohort (P5).

Participants expressed an appetite for further education around MH conditions and the care of patients with MH disorders. Suggestions included dual training, common conditions and their management, risk assessment, law and consent and physical restraint. This training might take the form of study days, part of the post‐registration critical care course or ad hoc bedside training delivered by an identified champion. However, it was acknowledged that the topic was vast, and providing education would be challenging:

So where do we start with the training and how much training do we need and how much can we stretch ourselves? Let's be honest. (P5).

In addition, there was concern that it would skills would not *stick* (P10) if they were not used regularly, alongside a feeling that the ICU nursing role is becoming impossibly wide:

The sort of acuity in the hospitals and the expectations and the feasibility versus the actual reality of what's achievable is all out of measurement and out of line. (P4).

**Theme 3**: Perceptions of patients with a MH disorder in ICU: **‘**people don't recognise that it is an illness**’**.

Patients with MH disorders were described as challenging and time consuming. One participant noted that if a nurse has two patients and one had an MH disorder, then the ‘other one gets neglected*’* (P2). Another participant commented that the general perception of MH patients is that they require ‘a lot of interaction*’* (P6). Some described MH patients as manipulative:

*…*will sort of say, well, if you don't do that, I'm going to go and kill myself. They use it as a bargaining tool (P4).

Such patients were perceived by staff as an unpopular allocation, particularly if they were a long‐stay admission who had engaged in deliberate self‐harm:

It's really sad, but some people see the difficulty they must have been going through to get to that position, whereas others just don't care. They would rather that, you know, they been successful in their attempt (P12).

In contrast, many participants described inclusive and equitable practice, emphasising that MH is part of critical care nursing (P12). Other participants mentioned how personal or familial experiences with MH disorders had increased the level of compassion with which they viewed this patient group:

How do you feel about depersonalising and dehumanising them now? Because that could have been you (P10).

When discussing empathy, participants seemed to focus specifically on patients admitted following suicide attempts. In contrast to comments about this being a potentially manipulative action, other participants saw is as the result of ‘years seeking help and failing*’* (P11):

This person is not taking the piss. They are being tortured emotionally, psychologically, circumstantially, and I need you to understand that this is their way of dealing with indescribable pain (P11).

Participants described the need to *model* (P11) behaviours to colleagues to generate empathy and understanding for patients with MH disorders. They countered stigma through encouraging nursing staff to put themselves in the position of their patients, emphasising that MH disorders can be as serious as physiological illnesses:

But would I want to come into ICU with three organs failing? No, I don't. But would I want to come in because I felt so bad that day that I tried to kill myself? No, I wouldn't. (P10).

**Theme 4**: Unpredictable and violent: ‘No‐one knows what to do with a mental health patient in ICU’.

The behaviour of patients with an MH disorder was described as inherently unpredictable. This caused nurses to feel *on edge* (P2) as ‘something completely innocent’ (P9) could cause a behavioural escalation: ‘And boom, just like that. They're gone’ (P10). Support from mental health services was described as *comforting* (P2), especially if they were able to describe potential behaviours to the ICU staff. *Impulsive behaviour* (P10) was challenging for ICU nurses as they lacked ‘a very clear point of control or a point of interventions’ (P5). This was seen as a contrast to physiological unpredictability which could be manipulated by drug administration: ‘ICU is all about control*’* (P5). To guard against rapid changes in behaviour and preserve their own safety, nurses felt that they must closely supervise their patient:

*…*like making sure that you're safe. So, you have like exit points, making sure that you can kind of see the person at all times. (P1)

Positioning MH patients safely on the unit was also described as *difficult* (P4), with both bays and single rooms seen as having safety risks. However, the close observation and anticipation of risk was deemed by one participant to have a negative impact on the level of compassionate care provided (P10).

Participants found the absence of clear clinical guidance for the management of patients with a mental health disorder on ICU increased the challenge of nursing this patient group. One participant recalled being requested by a member of medical staff to locate the rapid tranquilisation policy only to find that ‘the whole trust has no rapid tranquilisation policy’ (P11). It was noted that the presence of a pathway could guard against medications being omitted and ensure that the ICU had appropriate resources in place prior to admitting a patient with an MH disorder, but acknowledged the challenge of developing a clinical pathway for such a heterogeneous population:

*…*and that we would agree a set of adjuncts with mental health personnel to that would be useful in situ in such situations. But, you know, mental health presentation conditions being many and varied that would be difficult to, you know, hone down to a regular set of meds*…* (P8).

Access to timely support when managing violent behaviour was difficult:

We'll call security, but again, inherently it will be at least 10 min before they turn up in which by which point we'll often have brought the situation to some form of control*…* they'll stay for a limited period of time for what they consider to be, you know, an acute episode. Once that's settled down there, they'll be off (P8).

A number of participants described their experiences of violent behaviours:

And he grabbed my fingers and actually broke one of my fingers and damaged ligaments and tendons in another one (P12).

Both physical and psychological injuries were described. One participant recalled a colleague whose patient became acutely agitated:

It was scary. The fact that we had security guards, we had nursing staff pinning this patient down. We assaulted this patient, in want of better the word, for his own benefit because he was unsafe and he could have killed himself in that state (P3).

That nurse went on to refuse to care for further patients with MH disorders. Violent incidents were described as making junior nurses ‘back away in sheer horror’ (P8) and were associated with staff needed to take sick leave to ‘process what's happened’ (P8). A further participant appeared desensitised to the violence:

Yeah, yeah, I got kicked in the face. Who hasn't been injured by a mental health patient? (P10).

Support following assault was described as insubstantial:

I just didn't feel like work supported me and that I don't think they really thought about the impact that had on me (P12).

**Theme 5**: The ICU environment is unsuitable for MH patients: ‘but we're deemed the safest place to send them’.

The long‐term and challenging nature of MH disorders was seen as incongruous with the essence of critical care. In contrast to physiological disorders, where nurses can ‘give this to fix this and that’ (P10) and which were deemed ‘things we can change very, very easily’ (P3), MH was seen as an *unsolvable mire* (P10):

We can keep your airway patent, and we can stop you aspirating and, you know, and treat your poisons that you've took, but we can't fix your head (P10).

The critical care environment was also considered inappropriate for patients with MH disorders. Participants considered how the environment might present itself to patients admitted following deliberate self‐harm:

Watching other people come in extremely unwell, intubated and ventilated, you know, with curtains around, but privy to bleeding, screaming, crash calls at 4:00 AM, and if this person already had really significant distress, kind of essentially, in their experience, holding them captive in hospital in witnessing horrible things (P11).

Participants acknowledged that ICU is noisy and frightening (P4) and that the bright lights and staff dressed identically in scrubs (P9) can contribute to a sense of overwhelm and isolation (P12). The presence of ‘all these people trying to die’ (P10) was considered especially distressing for survivors of suicide attempts.

The critical care environment was also considered unsuitable in terms of the care which could be delivered. Participants noted that MH patients lacked specific therapeutic care while being nursed in ICU and that there was a tendency to ‘put a plaster on and then send them out back into the big wide world’ (P1) rather than address the underlying problems which led to admission:

Wake him up. Get them medically fit, and then we can get mental health liaison and pretty much, that's all we do. We don't engage in any other therapeutic intervention with them once they're with us (P10).

ICU was described as a *no‐man's land* (P4) where MH teams were perceived as not wishing to take responsibility for the patients. In addition, ICU appeared to be regarded as a safe place, due to the high nurse to patient ratio. These factors contributed to patients staying on critical care after they were medically fit for discharge (P5). However, one participant noted that the high staff ratio on ICU was detrimental:

*…*but this patient probably enjoyed attention, shall we say, and was really delighted to be on ICU and to be receiving this one‐on‐one medical attention. (P7).

The view that ICU was a safe environment for MH patients was contested by several participants. A bed space required careful preparation prior to admitting or waking a patient with an MH disorder. Needles, drugs, oxygen tubing and other adjuncts were all considered potential risks:

If it's something that we can get rid of, we'll get rid of it. (P1).

However, it was noted that not everything could be safely removed, and also that, as a critical care area, there was an expectation that adjuncts which would support any physiological deterioration would be easily accessible for the nurse:

*…*if you're in an intensive care bed and you've got potential to deteriorate or to become more unwell*…* you need to have equipment there. And actually, if we've just stripped this bed space because the risk, well, should she be here? (P4).

ICU was described as ‘the worst place in the hospital’ (P10) to have a ‘mental health patient walking around’ (P10). They were considered a risk to other critical care patients and their relatives. Acts of verbal or physical aggression were deemed to be *a problem* (P8) as they could cause distress to other patients and relatives. MH patients were also considered to be a physical risk to the safe delivery of critical care:

*…*we're in the next bed space from somebody who is properly critically ill and if this patient becomes in any way involved in the ongoing renal filtration, intubation, ventilation, you know, heart pump situation, that's going to be really, really messy (P11).

## 4 Discussion

This study offers insight into the experiences of ICU nurses when managing a patient with an MH disorder. Five themes were identified which demonstrated concerns about the integration of physical and MH care, a perceived lack of knowledge, the impact of violence and aggression and frustration that ICU was regarded as a safe and suitable place to nurse a patient with an MH disorder.

NICE [21] emphasise the importance of rapid psychosocial assessment by an MH professional following admission for self‐harm. However, participants described delays in assessment due to poor staffing and high workloads and difficulties in accessing support. One participant described a delayed assessment causing distress to their patient. Despite government plans to increase equity of access to MH liaison services in acute hospital settings [22], participants found such services to be challenging to contact and slow to respond to requests for support. The separation of mental and physical health care can cause patient distress and disrupt their treatment plan [23].

In line with recent reviews [15, 16], participants felt that they lacked knowledge about the care and management of patients with an MH disorder, but acknowledged that this is a vast topic which would be challenging to combine with existing ICU competencies. MH care is not included in the UK Steps Competency Framework for critical care nurses [24] nor the Guidelines for the Provision of Intensive Care Services [25]. A lack of physical restraint training was also identified. This was surprising because restraint is common practice in critical care and is used to maintain patient, device and staff safety [26]. Despite expressing discomfort [26, 27], nurses did initiate restrictive management when they felt it was necessary. This contrasts with some participants in this study who felt that managing escalating aggression or agitation in the case of an MH patient should be undertaken by an MH nurse or trained security. There appeared to be a sense that MH patients are not ICU patients and require different care and management. This could impact negatively on ICU nurses' ability to use their existing skills, many of which are transferable to this patient cohort.

Socially constructed stigma about MH disorders, and especially patients who self‐harm or attempt suicide, can lead patients to be marginalised or excluded from care [7, 28]. Participants expressed reluctance and discomfort about engaging with this patient group. Similar attitudes have been found among Emergency Department nurses [29]. Unwillingness to communicate openly about self‐harm is associated with impaired nurse–patient relationships and the risk of further harm [29, 30]. It is therefore important that ICU nurses are supported to develop skills in this area of communication. Patients who had attempted suicide or self‐harmed were at the forefront of many participants' recollections, and such a patient formed one of the fictitious case studies used in some interviews. Survival of critical illness is itself associated with an increased risk of self‐harm and suicide, and this risk is further increased with the presence of a pre‐existing MH disorder [31]. Maiden et al. [32] reviewed 750 cases admitted to ICU following self‐harm. Of these, 24 died within a year of discharge. Half of these deaths were caused by further self‐harm. There is a paucity of evidence on the experience of critical care by patients who self‐harm or attempt suicide.

Participants expressed concern about the safety and suitability of ICU as an environment in which to nurse MH patients. Some concerns were associated with the presence of multiple sharps risks and potential ligature points. However, Mills, Watts and Hemphill [33] found only seven suicide attempts by ICU patients in their review of 525 recorded attempts between 1999 and 2012 in US Veterans' hospitals. The authors suggested that staff should be vigilant and reduce patient access to sources of harm, seek psychiatric input and consider support post‐discharge in case of further suicidal ideation [33].

## 5 Limitations

This study is subject to potential social desirability bias due to its topic. Participants volunteered to join the study, suggesting that they might have a prior interest in MH, or that they had experienced something clinically which they were keen to discuss. This, together with the small heterogeneous sample size, may limit the transferability of the results. Member checking was not undertaken as the researchers did not wish to add further to participants' workloads, and additionally because evidence suggests that member checking may not enhance validity [34]. A reflexive approach to analysis was followed [19, 35]. This approach reframes researcher subjectivity from a weakness and, instead, recognises its value and encourages authors to reflect upon their own perspectives and experiences as they undertake analysis.

## 6 Implications for Practice and Further Research

This study adds to a growing body of knowledge around how patients with an MH disorder are managed in critical care. As with previous reviews [15, 16], there are concerns that ICU nurses lack knowledge around how to care for MH patients and are therefore at risk of providing care based in social stigma and stereotypes. Negative memories of challenging patients have been shown to have a lasting impact on the way staff perceive MH patients [14]. There is a need for strong leadership and support for staff affected by violence and aggression.

Participants described conflict and frustration with their MH colleagues. It appeared that there were misunderstandings and a lack of parity of esteem [36]. In order to deliver safe, effective and timely MH support, it is vital that teams work together [21]. Education at both pre‐ and post‐registration levels, together with inter‐professional learning opportunities, could lead to a more cohesive approach to the care of MH patients outside of psychiatric settings [37].

This study is part of an on‐going research project. Following on from the concern participants expressed in these interviews regarding patients admitted following self‐harm or attempted suicide, the next study will seek to explore the experience of this patient group on ICU. Patient voices have been shown to be effective in teaching and anti‐stigma programmes [38, 39], and it is hoped that the results of these studies will support critical care nurses in providing holistic care to patients with MH disorders.

## 7 Conclusions

This study has demonstrated that despite being regularly tasked with caring for patients with a MH disorder, ICU nurses find this cohort challenging and feel that they lack knowledge and support to manage them effectively. Participants described a lack of cohesion with MH services and poor support following episodes of violence and aggression. The results of this study indicate a need for increased clinical support and education, and a more collaborative working relationship with MH services to ensure the delivery of timely and appropriate care. Participants found communication with patients who have self‐harmed or attempted suicide especially challenging. Future research is planned to explore the lived experience of this patient group to inform staff education and improve patient support.

## Funding

The authors have nothing to report.

## Ethics Statement

This study was given favourable review by the University of Leeds School of Healthcare Research Ethics Committee (ID: 2755) in May 2025.

## Consent

Informed consent was obtained from each participant.

## Conflicts of Interest

The authors declare no conflicts of interest.

## Data Availability

Data are not available due to confidentiality as participants may be identifiable.
